# Tracking Bacterial Nanocellulose in Animal Tissues by Fluorescence Microscopy

**DOI:** 10.3390/nano12152605

**Published:** 2022-07-28

**Authors:** Renato Mota, Ana Cristina Rodrigues, Ricardo Silva-Carvalho, Lígia Costa, Daniela Martins, Paula Sampaio, Fernando Dourado, Miguel Gama

**Affiliations:** 1 CEB—Centre of Biological Engineering, University of Minho, Campus Gualtar, 4710-057 Braga, Portugal; renatovelosomota@gmail.com (R.M.); anacris38599@gmail.com (A.C.R.); remanuelcarvalho@gmail.com (R.S.-C.); lfr.costa92@gmail.com (L.C.); dsr.martins92@gmail.com (D.M.); fdourado@deb.uminho.pt (F.D.); 2 LABBELS—Associate Laboratory, 4710-057 Braga, Portugal; 3 Instituto de Investigação e Inovação em Saúde, Universidade do Porto, 4200-135 Porto, Portugal; sampaio@ibmc.up.pt; 4 IBMC—Instituto de Biologia Celular e Molecular, 4200-135 Porto, Portugal

**Keywords:** food additive, bacterial nanocellulose, bacterial cellulose nanocrystals, cellulose binding module, fluorescence microscopy, gastrointestinal tract, absorption

## Abstract

The potential of nanomaterials in food technology is nowadays well-established. However, their commercial use requires a careful risk assessment, in particular concerning the fate of nanomaterials in the human body. Bacterial nanocellulose (BNC), a nanofibrillar polysaccharide, has been used as a food product for many years in Asia. However, given its nano-character, several toxicological studies must be performed, according to the European Food Safety Agency’s guidance. Those should especially answer the question of whether nanoparticulate cellulose is absorbed in the gastrointestinal tract. This raises the need to develop a screening technique capable of detecting isolated nanosized particles in biological tissues. Herein, the potential of a cellulose-binding module fused to a green fluorescent protein (GFP–CBM) to detect single bacterial cellulose nanocrystals (BCNC) obtained by acid hydrolysis was assessed. Adsorption studies were performed to characterize the interaction of GFP–CBM with BNC and BCNC. Correlative electron light microscopy was used to demonstrate that isolated BCNC may be detected by fluorescence microscopy. The uptake of BCNC by macrophages was also assessed. Finally, an exploratory 21-day repeated-dose study was performed, wherein Wistar rats were fed daily with BNC. The presence of BNC or BCNC throughout the GIT was observed only in the intestinal lumen, suggesting that cellulose particles were not absorbed. While a more comprehensive toxicological study is necessary, these results strengthen the idea that BNC can be considered a safe food additive.

## 1. Introduction

The 21st century’s environmental and economic challenges, in particular related to sustainability and safety, have been driving the preference for the use of green, renewable and recyclable raw materials for the production of high-value-added products but with lower environmental impact [1]. Nanocellulose (NC), a nano-scaled cellulosic material (with at least one dimension <100 nm), from plant or bacterial sources [1,2,3,4], is at the forefront of such promising biopolymers [2]. Due to its large specific area, negligible toxicity, low density, biodegradability and biocompatibility, NC has increasingly been explored in several fields such as textiles, pharmaceuticals, cosmetics, medicine, food and packaging [4,5,6]. In 2020, the worldwide market for NC has been estimated at USD 297 million and is expected to increase by 2025 to as much as USD 783 million [7].

As food additives, micron and colloidal plant cellulose and their derivatives are approved for human consumption by several regulatory authorities, such as the FDA (Food and Drug Administration, Silver Spring, MD, USA) and the EC (European Commission); they are regularly applied in the food industry to regulate its texture, stability, rheology and organoleptic features [8,9]. In the 1980s, plant nanocellulose was first suggested as a human food additive with enormous potential, as its high aspect ratio could confer new physicochemical properties and behaviors to foods [10]. Ever since, several other studies have pointed out the potential of NC to further improve food quality and appeal, as well as their capacity to modulate digestion and nutrient absorption [11]. But, because of their nanoscalar nature, different properties and interactions with biological systems may arise, as compared to the micro-sized counterparts [12,13], raising several concerns throughout the various stages of NC life cycle. This is especially relevant, as the biological effects of nanocelluloses depend on their chemical nature, size, shape, aggregation properties, degree of branching, specific surface properties, among others. These intrinsic properties of NC, affecting their interactions with cells and living organisms, are still poorly understood [14,15,16]. For instance, nanotoxicity derives mainly from the small size and large surface area of engineered nanomaterials, which may enable their translocation to different organs by getting absorbed into the blood through the intestinal lumen [17,18,19]. Several publications have reported the toxicity of ingested inorganic nanomaterials such Ag or ZnO nanoparticles in animal models [20,21,22]. Regarding plant or bacterial celluloses’ absorption in the gastrointestinal tract (GIT), few and contradictory studies have been published [23,24,25,26,27,28]. Reports have shown that bacterial [23] and plant cellulose [8] are slowly and limitedly degraded in rats’ large intestine, yielding metabolites that are partially absorbed by the colon and/or microflora, in both cases used mainly as an energy source. These cellulose degradation products, absorbed in the intestine, were detected in urine and in exhaled CO_2_. Studies with germ-free rats (no intestinal microflora) were also carried out, wherein the total excretion of cellulose was observed, thereby concluding that there is no absorption of undegraded cellulose but only of its degradation products [23]. Thus, a thorough environmental and risk assessment (the fate and toxicity of NC in the human body) is of extreme importance when considering NC production (occupational exposure), commercialization and use (human consumption).

On the other hand, the lack of appropriate detection and characterization techniques and the absence of reproducible and validated methods for toxicological studies have been identified as major bottlenecks in the evaluation of the safety of nanomaterials [12,13,29,30,31,32]. For instance, the metabolization of cellulose by the colon microflora makes it very difficult to distinguish the celluloses’ fibers from their degradation products, not allowing the clarification of whether the fibers are absorbed in the GIT. BNC is a nanofibrillar exopolysaccharide produced by acetic-acid-bacteria, such as the ones from the genera *Komagataeibacter*. Although chemically identical to plant cellulose, BNC is chemically pure and has higher tensile strength, water-holding capacity and crystallinity than cellulose from plant sources [4]. Furthermore, while plant cellulose comminution to the nanoscale requires mechanical, chemical or enzymatic processes, BNC is naturally nano-sized [4,9]. In food applications, BNC is marketed mostly in Asia as “nata de coco” [4,9,33], but it has been attracting the attention of many industries worldwide, given its unique features. The available toxicological data on BNC has been extensively reviewed by Dourado et al. (2016), wherein (i) the absence of genotoxicity, carcinogenicity, pyrogenicity or developmental or reproductive toxicity and (ii) the long history of its consumption (without any reported cases of toxicity) were exposed [33]. Like plant nanocellulose, the potential hazards of ingested BNC, derived from its nanoscale nature, are insufficiently characterized, hindering its entry into the Western food market.

Fluorescence-based detection is the most common method used in biosensing, due to its relatively low-cost and high sensitivity, specificity and simplicity. On the other hand, electron microscopy is the technique of choice when a high resolution is required [34,35,36,37]. To fill the gap between light and electron microscopies, correlative light and electron microscopy (CLEM) strategies have been developed, allowing the establishment of a correlation between a particular ultrastructural feature (by SEM or TEM) and a fluorescence signal [34,35,36,37]. This approach could be used to determine the sensitivity of fluorescence microscopy (FM) and its potential to detect cellulose nanostructures in biological tissues, namely in the GIT, in particular BNC. Thus, the present study was designed to evaluate whether fluorescence analysis is sensitive enough to detect isolated bacterial cellulose nanocrystals (BCNC) using CLEM. As such, we prepared BCNC by acid hydrolysis [38,39,40] to simulate an (unlikely) scenario of the extreme digestion of BNC in the GIT. The BCNC were stained with a fluorescently labeled cellulose-binding domain [41], and, using CLEM, we attempted to determine whether isolated nanocrystals are detected by FM. After such validation, fluorescence microscopy was used in the analysis of the histological slides of different tissues from Wistar rats, fed with BNC, to determine whether the fibers (or their degraded versions) could be detected, establishing a platform for more comprehensive studies.

## 2. Materials and Methods

### 2.1. Materials

BNC membranes were obtained from HTK Food Co., Ltd. (Ho Chi Minh City, Vietnam). Chemicals used for the reaction of the bacterial cellulose nanocrystals, namely H_2_SO_4_ and HCl, were supplied by Thermo Fisher Scientific (Waltham, MA, USA).

### 2.2. Methods

#### 2.2.1. Preparation of Bacterial Nanocellulose

The as-received BNC membranes were washed with distilled water (dH_2_O) until the pH became that of the dH_2_O, to remove any soluble chemicals. Then, the membranes were cut into small pieces and ground using a high-speed blender (Moulinex Ultrabend1 500 W, Écully, France), at 24 000 rpm for 5 min, to obtain a pulp, which was then filtered. The BNC cake was further concentrated by centrifugation at 11 000 rpm (Centrifuge 5430 R, Eppendorf, Hamburg, Germany) for 20 min at room temperature (RT). The solid fraction of the centrifugate was adjusted to 10% (*m/v*) with dH_2_O and stored at 4 °C in a glass container until use.

#### 2.2.2. Production of Bacterial Cellulose Nanocrystals

The BNC centrifugate was subjected to acid hydrolysis with a solution of H_2_SO_4_/HCl (34 and 24% *m/m*, respectively), as described elsewhere [39]. For each batch of BCNC production, 10 g of BNC (at 10% solids) and 1 000 mL of acid solution were mixed at 45 °C with magnetic stirring (500 rpm) with a H03D mini-stirrer (lbx Instruments, Lonay, Switzerland) for 75 min. The hydrolysis reaction was then stopped by diluting the reaction 15-fold with cold dH_2_O. The suspension was ultracentrifugated at 11 000 rpm (Heraeus Multifuge X3R, Thermo Fisher Scientific, Waltham, MA, USA) for 15 min at RT to precipitate the BCNC, which were then washed with dH_2_O under several cycles of ultracentrifugation (11 000 rpm for 20 min) to remove excess acid. This procedure was repeated until the pH was in the range 5–7. The resulting suspension was sonicated for 3 min at 500 W (Sonics & Materials, Newtown, CT, USA), and the final concentration was adjusted to ∼1% (*m/v*).

### 2.3. Characterization of the BCNC

#### 2.3.1. Zeta Potential

The stability of the BCNC aqueous suspension was assessed by measuring the particles’ surface zeta potential. For that, BCNC suspensions of 0.1% (*m/v*) in dH_2_O were prepared and the zeta potential measured using a Zetasizer Nano ZS (Malvern Instruments, Malvern, UK). Data were calculated considering the viscosity of water at 25 °C of 0.893 ×10^−3^ Pa.s^−1^. Three measurements of each suspension were performed. Throughout the work, six batches of BCNC were made. The zeta potential result displayed is the average of all measurements.

#### 2.3.2. Fourier-Transform Infrared Spectroscopy (FTIR)

FTIR spectroscopic analysis of BNC and BCNC was carried out in a Bruker FTIR spectrometer ALPHA II (Bruker Corporation, Billerica, MA, USA) in transmission mode, operating at a resolution of 4 cm^−1^. BNC and BCNC were both frozen and processed in a freeze-drier (Coolsafe 100-9 Pro, Labogene, Allerød, Denmark). Two milligrams of the samples were then mixed with 200 mg of dry potassium bromide (Thermo Fisher Scientific, Waltham, MA, USA) to obtain a solid pellet that was scanned three times to check the authenticity of data. The spectra were taken between 4 000 and 700 cm^−1^ by averaging 24 scans for each spectrum.

#### 2.3.3. Transmission Electron Microscopy (TEM)

The morphology of the BCNC was assessed by TEM imaging. Briefly, 5 µL of BCNC aqueous suspension at 0.01% (*m/v*) was applied on the grid (FCF400-Cu, Electron Microscopy Sciences (EMS), Hatfield, UK) and allowed to settle for 2 min at RT. The sample was then blotted off, and 5 µL of uranyl acetate (UA) (Sigma-Aldrich, St. Louis, MO, USA) for negative staining was directly applied. The excess solution was again blotted off and replaced by another 5 µL of UA. Each time, the UA was allowed to incubate for 30 s before it was removed. The sample was observed with a JEOL 2100 plus TEM device (JEOL, Tokyo, Japan), operated at 80 kV accelerating voltage. Several images were taken, considering areas far from each other and trying to represent the whole grid’s surface. The length (L) and width (W) of the BCNC were determined from at least 150 measurements by image analysis, using ImageJ software (Version 1.51j8, Bethesda, MD, USA) [42].

### 2.4. GFP-CBM3A Adsorption onto BNC and BCNC

Knudsen et al. (2015) developed a method for the specific and sensitive detection of cellulose fibers, including nanofibrillar cellulose, in biological tissues (both in cryopreserved and paraffin-embedded tissues), using a biotinylated carbohydrate-binding module (CBM) of the β-1,4-glycanase from the bacterium *Cellulomonas fimi* [41]. Based on their work, we evaluated the efficiency of a GFP-fused CBM derived from *Clostridium cellulolyticum* (GFP–CBM3A, NZytech, Lisbon, Portugal) to bind both to BNC and BCNC. The recombinant GFP–CBM3A (henceforward designated as GFP–CBM, for the sake of simplicity) was purified from *Escherichia coli* and contains a family 3A carbohydrate-binding module (CBM3A) and an N-terminal green fluorescent protein (GFP). CBM from the type 3A family binds specifically to crystalline forms of cellulose [41].

For these experiments, a HORIBA scientific spectrofluorometer (Kyoto, Japan) was used, operating at emission and excitation wavelengths of 510 and 475 nm, respectively. A calibration curve using aqueous solutions of GFP–CBM at 0.005, 0.010, 0.0150, 0.020 and 0.025 mg/mL was first obtained.

Next, in eppendorf tubes, different solutions of GFP–CBM, with concentrations of 0.500, 0.250, 0.175, 0.100, 0.050, 0.025, 0.0125, 0.005 and 0.000 mg/mL in the final mixture, were each incubated with a fixed mass of 0.25 mg (dry basis) of BNC or BCNC in 0.40 mL of dH_2_O for 2 h (sufficient time to reach equilibrium, as observed in exploratory assays) at RT. Then, the dispersions were centrifuged (Centrifuge 5430 R, Eppendorf, Hamburg, Germany) for 10 min at 8 000× *g*. The GFP–CBM in the collected supernatant was quantified through spectrofluorometry, as described above.

A non-linear regression analysis was used to calculate the parameters of the Langmuir adsorption isotherm [43,44]:

(1) $${\mathrm{GFP}-\mathrm{CBM}}_{\mathrm{Bound}}=\frac{{\mathrm{GFP}-\mathrm{CBM}}_{\mathrm{Max}}\;.{\;K}_{a}\;.{\mathrm{GFP}-\mathrm{CBM}}_{\mathrm{Free}}}{1+{\;K}_{a}\;.{\mathrm{GFP}-\mathrm{CBM}}_{\mathrm{Free}}}$$

where GFP–CBM_Bound_ is the amount of adsorbed protein per unit mass of cellulose (mg/mg), GFP–CBM_Free_ is the protein concentration (mg/mL) in the liquid phase at the adsorption equilibrium, GFP–CBM_Max_ is the maximum amount of adsorbed protein per unit mass of cellulose (mg/mg), and K_a_ is the Langmuir constant (mL/mg). Three independent assays were performed, each of them in triplicate. The non-linear regression and parameters (K_a_ and GFP–CBM_Max_) were calculated using OriginPro 2018 software (Version 9.5.1.195, Northampton, MA, USA [45].

Finally, the binding of the GFP–CBM was also assayed qualitatively by the observation of the GFP–CBM-bound celluloses by FM on an Olympus BX51 (Olympus, Tokyo, Japan) with a 60×objective and a FITC filter set.

### 2.5. Correlative Light Electron Microscopy (CLEM)

The feasibility of FM to detect individual nanometric particles was evaluated by CLEM. To this end, reference grids (50/B D300F1FC-50CU, EMS) with adsorbed BCNC were observed using widefield fluorescence microscopy and then by TEM to detect co-localized single BNC crystals. Briefly, suspensions of BCNC were diluted to 0.001 mg/mL and labeled with GFP–CBM at a final concentration of 0.05 mg/mL for 30 min at RT. Next, 5 µL of GF–-CBM:BCNC suspension was applied to the grid and allowed to settle for 2 min. Then, the sample was blotted off and sequentially observed on (i) a FM Nikon ECLIPSE Ti (Nikon, Tokyo, Japan) with a 10x/0.45 or 40x/0.95 PlanApo objectives, FITC filter set and an IRIS 9 camera (Photometrics, Huntington Beach, CA, USA) under the control of NIS-Elements software (Version 4.5, Nikon, Tokyo, Japan) and then on (ii) a JEOL JEM 1400 Transmission Electron Microscope (JEOL, Tokyo, Japan). Images were digitally recorded using a CCD digital camera 1100W (Orius, Tokyo, Japan).

Some technical issues regarding sample throughput between FM and TEM observations arose, which were overcome by using gelatin from edible grade (Results and Discussion, Section 3.3). The effect of the gelatin matrix on the visualization of BCNC was first assessed in both FM and TEM. After optimization, 0.002 mg/mL BCNC labeled with GFP–CBM (0.05 mg/mL) (prepared as described above) were mixed with a solution of 2% gelatin (1:1 final mass ratio) for 15 min at 45 °C. After cooling to RT, the suspension was i) placed on microscope slides (VWR, Radnor, PA, USA) for FM evaluation or ii) applied to the grid (FCF200-NI-TA, EMS) and allowed to settle for 2 min before analysis. The length (L) and width (W) of the BCNC were determined using ImageJ (Version 1.51j8, Bethesda, MD, USA). Samples were processed for CLEM following the aforementioned methodology.

### 2.6. In Vitro Assays

#### 2.6.1. Cell Line and Cell Culture

Mouse fibroblast L929 cell line was purchased from American Type Culture Collection (ATCC, Manassas, VA, USA). The cell line was maintained in Dulbecco’s modified essential medium (DMEM) (Sigma-Aldrich, St. Louis, MO, USA) supplemented with 10% (*v/v*) iFBS (Sigma-Aldrich, St. Louis, MO, USA) and 1% (*v/v*) penicillin (10 000 U/mL, Sigma-Aldrich, St. Louis, MO, USA)–streptomycin (10 000 μg/mL, Invitrogen, Waltham, MA, USA) (complete DMEM medium) at 37 °C and 5% CO_2_.

#### 2.6.2. Bone-Marrow-Derived Macrophage (BMMΦ) Differentiation

Mouse bone marrow cells were differentiated in vitro as described elsewhere [46]. C57BL/6 mice were anesthetized using isoflurane and euthanized by cervical dislocation. Femurs and tibias were removed and cleaned in aseptic conditions. Bones were disconnected by the articulations and then flushed using complete DMEM medium. The obtained cell suspension of bone marrow cells was centrifuged (300× *g*, 10 min) at RT and the pellet resuspended in 15 mL of complete DMEM medium supplemented with 20% (*v/v*) L929-cell conditioned medium (LCCM) as a source of macrophage colony-stimulating factor (M-CSF). Prior to this, LCCM was prepared as follows: L929 cells (at a initial density of 5 × 10^3^ cells/mL) were grown for 8 days at 37 °C in a 5% CO_2_ atmosphere. Cells were then centrifuged (300× *g*, 10 min), the supernatant collected, filtered (0.2 µm filter) and finally stored at −20 °C before use. The bone marrow cell suspension was cultured overnight at 37 °C in a 5% CO_2_ atmosphere. The non-adherent cells were collected with warm complete DMEM medium supplemented with 20% (*v/v*) LCCM, plated in a 96-well plate and incubated once again at 37 °C in a 5% CO_2_ atmosphere. On the 4th and 7th days, half of the medium was renewed, and on the 10th day, cells became fully differentiated into macrophages.

#### 2.6.3. Cytotoxicity Testing

The effect of BCNC on the viability of BMMΦ primary cells and in L929 cells was studied using a standard resazurin assay [47]. Briefly, a monolayer of BMMΦ (at a density of 3 × 10^4^ cells/well) and L929 (at a density of 1 × 10^4^ cells/well) were each incubated for 24 h (37 °C in a 5% CO_2_ atmosphere) with increasing concentrations of BCNC (0.00, 0.001, 0.005, 0.01, 0.02, 0.04, 0.10, 0.20 and 1.00 mg/mL), previously sterilized by UV radiation for 15 min at RT. A higher BCNC concentration (2.0 mg/mL) was also used for cytotoxicity evaluation in BMMΦ cells. After incubation, 10% (*v/v*) of a 2.5 mM resazurin solution (Sigma-Aldrich) was added to each well, and the cells were incubated at 37 °C in a 5% CO_2_ atmosphere for another 4 h. A well containing only LCCM-supplemented DMEM medium was used as the blank sample. Fluorescence was measured (λex 560/λem 590 nm) in a SpectraMAX GeminiXS microplate reader (Molecular Devices LLC, San Jose, CA, USA). Results were expressed as the mean percentage ± SD of viable cells relative to a control without BCNC (considered as 100% viability). Three independent assays were performed, each in triplicate.

#### 2.6.4. Uptake of BCNC by BMMΦ Primary Cells

The uptake of BCNC by macrophages was evaluated either by FM and TEM. For that, 1 mL of BMMΦ cells (at a density of 1 × 10^6^ cells/well) was incubated in 24-well plates (at 37 °C in a 5% CO_2_ atmosphere) with a suspension of previously sterilized nanocrystals at a concentration of 0.001 mg/mL for (i) 2, 4, 6 and 24 h for fluorescence analysis and for (ii) 4 h and 24 h for TEM analysis.

For FM, inside each well was a chemically modified glass coverslip (ECN 631-1578, VWR) that allowed the macrophages to grow and adhere to its surface. After each incubation period, the medium was removed, and the cells were rinsed in fresh medium and fixed for 15 min in 4% paraformaldehyde (PFA) (Sigma-Aldrich, St. Louis, MO, USA). Subsequently, cells were rinsed 3 times with PBS (Sigma-Aldrich, St. Louis, MO, USA), permeabilized with 0.1% Triton X-100 (PanReac, Barcelona, Spain) at RT for 15 min and rinsed again with PBS. Nuclei were stained with 1 µg/mL DAPI (Frilabo, Porto, Portugal) at RT for 30 min. Cells were rinsed again with PBS, and then the actin-cytoskeleton was stained with 1 µg/mL phalloidin-TRITC (pha-red) (Sigma-Aldrich, St. Louis, MO, USA) for 30 min at RT. Cells were rinsed with PBS and incubated with GFP–CBM (0.05 mg/mL) for BCNC detection. After 3 final rinses in PBS, the coverslips were removed from the wells using tweezers and mounted for FM analysis. As control, macrophages without BCNC incubation were used. FM images of the stained macrophages were acquired using an Olympus BX61Confocal Scanning Laser Microscope (Model FluoView 1000, Olympus, Tokyo, Japan) using the following combination of excitation/emission detection-range wavelengths: 405/430–470, 488/505–540, and 559/575–675, for the visualization of the cells’ nuclei, GFP–CBM:BCNC and cells’ cytoskeleton, respectively. Images were acquired with the software FV10-Ver4.1.1.5 (Olympus, Tokyo, Japan).

For electron microscopy analysis, the cells grew and adhered directly to the bottom of the well plate. After the incubation period, trypsin was used to detach the cells, which were centrifuged for 10 min at 800× *g*. The resulting pellet was fixed in a solution of 2.5% glutaraldehyde (#16316; EMS) with 2% formaldehyde (#15713; EMS) in 0.1 M sodium cacodylate buffer (pH 7.4) for 1 day and post fixed in 1% osmium tetroxide (#19190; Electron Microscopy Sciences, Hatfield, UK) and 1.5% potassium ferrocyanide (Sigma-Aldrich, St. Louis, MO, USA) diluted in 0.1 M sodium cacodylate buffer (Sigma-Aldrich, St. Louis, MO, USA). After centrifugation, the pellet was washed in dH_2_O and then stained with aqueous 1% UA solution overnight, dehydrated and embedded in Embed-812 resin (#14120; EMS). Ultra-thin sections (50 nm thickness) were cut on an RMC Ultramicrotome (RMC Boeckeler, Hamburg, Germany) using Diatome diamond knifes, mounted on mesh copper grids (EMS) and stained with UA substitute (#11000; EMS) and lead citrate (#11300; EMS) for 5 min each. As control, macrophages without BCNC incubation were used. Samples were viewed on the JEOL 1400 (Tokyo, Japan), and images were digitally recorded using a CCD digital camera (Orius 1100W, Tokyo, Japan).

### 2.7. Cellulose Tracking in Animal Tissues

#### 2.7.1. Animals, Housing and Feeding Conditions

The study was performed at I3S. The experimental procedures followed the EU Directive 2010/63/EU and National Decreto-Lei 113/2013 legislation for animal experimentation and welfare. The rats’ housing, handling and experimentation were accredited by the Portuguese National Authority for Animal Health, Direção-Geral de Alimentação e Veterinária (DGAV) (approval n°012910/2020-08-07).

This pilot study aimed at establishing the methodological grounds—namely concerning the detection of cellulose fibers by a histological analysis of the tissues collected from the rats—for a larger and more comprehensive study.

Eight-week-old Wistar Han IGS Rats (Crl:WI(Han)), four male and four female, were used in this study. The rats were obtained from Charles River (Barcelona, Spain) and bred at a i3S animal facility. On arrival, the animals were examined for signs of health, followed by a one-week adaptation period. They were kept under controlled environmental conditions for one week before starting the experiment. Each rat was uniquely numbered with a color marker in their tail and placed in polycarbonate type III H cages with a stainless-steel wire lid and a polysulphide filtertop cage (Tecniplast, West Chester, PA, USA), with corncob and carboard tubes as bedding materials. An artificial light/dark cycle with a sequence of 12 h was applied. The room was ventilated with about 15–20 air changes/h. A temperature of 22 ± 2 °C and a relative humidity of 55 ± 15% was maintained.

Feed and water were provided ad libitum to all animals during the experiment. They received a commercial 2014S Teklad rodent diet (Teklad diets, ENVIGO) based on 14.3% crude protein, 4% fat, 48% carbohydrates and 4.1% crude fiber in addition to vitamins and fatty acids.

#### 2.7.2. Test Substance and Dosing Concentration

Given the high viscosity of BNC aqueous suspensions, the concentration for daily gavage was adjusted to 1% (*m/v*), as measured by gravimetry after drying overnight at 105 °C, by adding dH_2_O. The ground BNC was sterilized by autoclaving for 20 min at 120 °C and 1 bar; after cooling to RT, the suspensions were stored at 4 °C under sterile conditions. Before feeding, the suspensions were warmed to RT and homogenized by vigorously vortex agitation.

#### 2.7.3. In Vivo Study

This study comprised one dose group (each animal received 0.75 mL of the previously prepared 1% BNC suspension daily for a 21-day period) of 4 animals (two male and two female). Oral gavage was performed in the morning at a fixed time, using a polypropylene gavage needle of 1.3 × 1.3 mm without a ball tip. Each animal was observed daily for clinical signs. As control, animals fed only with commercial feed for 21 days were used.

Following sacrifice, the animals’ intestinal tract was collected and processed by the Swiss roll technique (SRT). The small intestines were collected and cut into three equal segments to obtain the duodenum, jejunum and ileum regions. The lumen of each small/large intestine portion was washed with PBS, processed by the SRT, cut longitudinally, opened so that the lumen is facing upward and then rolled. All of these tissues were embedded in OCT compound (Tissue-Tek^®^, SakuraTM, Flemingweg, The Netherlands), frozen and cryo-sectioned in 20 µm-thick slices (LEICA CM 1900, Wetzlar, Germany).

#### 2.7.4. Staining and Microscopic Observations

All samples were subjected to UV pre-treatment and final Sudan Black B (SBB) (Sigma-Aldrich, St. Louis, MO, USA) staining to remove tissue autofluorescence that could hinder cellulose detection with fluorescent CBM [48]. Briefly, after samples’ pre-treatment with UV for 2 h, they were fixed with 4% PFA for 30 min, followed by a washing step with PBS buffer at RT. Cells’ permeabilization was carried out with 0.5% Triton X-100 in PBS at RT, in a humid chamber (HC), then the blocking step was performed with 10% (*v/v*) fetal calf serum (FCS) (Sigma-Aldrich) in PBS for 1 h. For nucleus visualization, the slides were incubated with DAPI (1 µg/mL) for 10 min. After washing, pha-red (1 µg/mL) was applied, for 30 min in a HC, to stain the actin cytoskeleton. Then, samples were washed with PBS (three times) and incubated with GFP–CBM (0.05 mg/mL) for 2 h at RT in a HC. Sections were washed again with PBS and incubated with 0.1% SBB for 20 min in HC. Finally, the slides were washed with PBS and mounted with permafluor mounting media (Sigma-Aldrich, St. Louis, MO, USA).

At last, FM images of the stained histological samples were acquired using the Oympus BX61 Confocal Scanning Laser Microscope (Tokyo, Japan).

### 2.8. Statistical Analysis

The obtained raw data were statistically analyzed using GraphPad Prism software (Version 8.0.2.263, Graph Pad Software, Inc, San Diego, CA, USA). Differences in data from cytotoxicity assay were analyzed statistically using one-way ANOVA, with Dunnett’s multiple comparison test. All of the treatment conditions were compared with the control, and a 95% level of confidence (*p* < 0.05) was used.

## 3. Results and Discussion

The extent of BNC degradation in the human microbiome as well as its fate in the human body are still poorly characterized. This represents a primary obstacle towards its use in food systems, given the current regulatory constraints on the use of nanomaterials. In this work, we first aimed at establishing the methodological tools that allow the detection of individual BCNC through fluorescent microscopy. For this, BNC was chemically hydrolyzed into its elemental structural unit (BCNC). CLEM was then used to demonstrate that the same isolated BNC nanocrystals identified by TEM could also be visualized by FM. Secondly, using FM, we aimed to evaluate the cellular uptake of BCNC by phagocytic cells. Finally, an exploratory in vivo study using Wistar rats was performed to evaluate the behavior of BNC along the GIT, namely its potential absorption.

### 3.1. BCNC Production and Characterization

The combination of sulfuric and hydrochloric acids has been shown to allow the production of stable BCNC with high dispersibility in aqueous suspensions [39]. In this work, the surface charge of the prepared BCNC was evaluated by means of Zeta-potential analysis. It is generally considered that colloidal systems with particles bearing a modulus of >30 mV reflect good stability due to electrostatic repulsion forces [49]. BCNC presented an average zeta value of −45.2 ± 0.7, indicative of such good stability. The highly negative surface charge density of the BCNC is due to the conjugated sulfate groups generated from the esterification of the hydroxyl groups at the surface of the BCNC, since HCl does not react with the hydroxyl groups [49,50,51].

**Figure 1:**
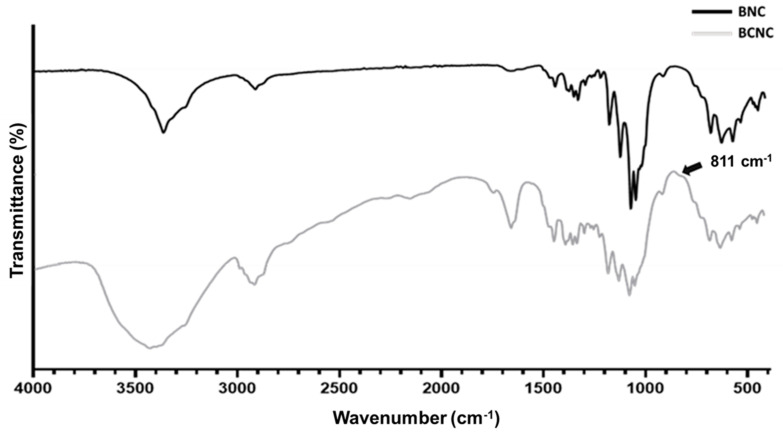
FTIR spectra of BNC and BCNC.

Figure 1 displays the FTIR spectra of BNC and BCNC. Both samples exhibited similar vibration bands, namely ~900, 1030–1165, 1375–1475, 1635, 2900 and 3350–3400 cm^−1^, well described in the literature: C-O-C stretching from β-(1,4) glycosidic linkages are attributed to the band near 900 cm^−1^; peaks ranging from 1030 to 1165 cm^−1^ have reflected other C-O bonds; the ones comprised between 1375–1435 cm^−1^ are assigned to C-H bending; the wide bands around 3350–3400 and 1630–1700 cm^−1^ were related to the O-H groups of cellulose; finally, the band at 2900 cm^−1^ represented C-H stretching [52]. As expected, the small shoulder at 811 cm^−1^ (black arrow), only observed in the BCNC spectra, corresponds to the C-O-S group vibration, owing to the establishment of sulfate esters on nanocrystal surfaces during the acid hydrolysis [52].

**Figure 2:**
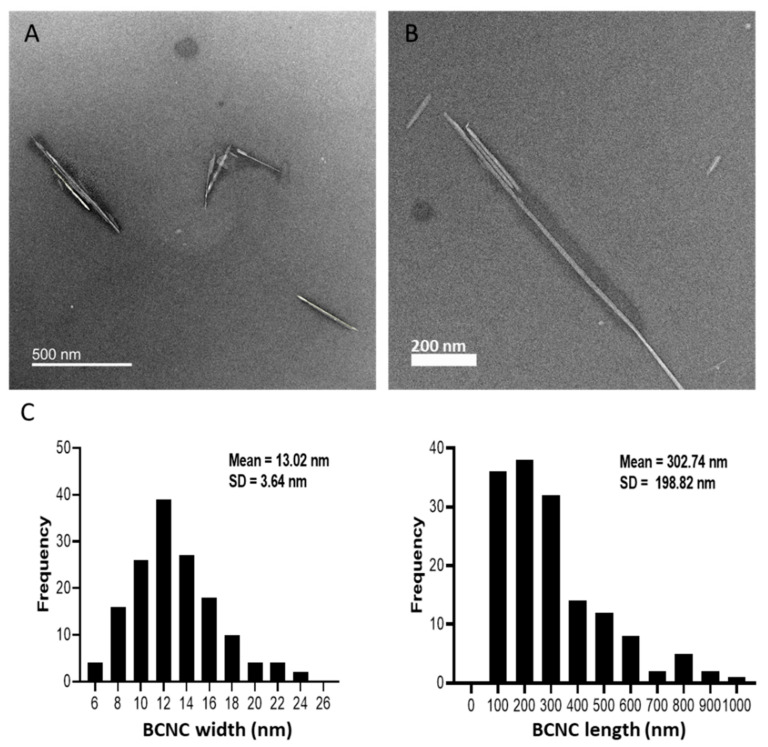
(**A**,**B**) TEM micrographs of the BCNC prepared by the acid hydrolysis of BNC and (**C**) the corresponding particle-size distributions. Scale bars: (**A**) 500 nm and (**B**) 200 nm.

With TEM imaging (Figure 2), BCNC display a typical needle-shaped structure [46,48], with dimensions of 6–25 nm in width (average W of 13 ± 4 nm) and about 50–1100 nm in length (average L of 303 ± 199 nm). The BCNC dimensions are close to those reported in the literature for BCNC prepared under similar hydrolysis conditions [39,41,49].

As could be expected from their high modular value of the zeta potential, any aggregates observed consisted of only few nanocrystals, mostly organized ‘side by side’. Similar size-distribution profiles were reported in other works, in particular for cellulose nanocrystals produced by HCl hydrolysis [53,54,55].

### 3.2. GFP–CBM Adsorption onto BNC and BCNC

**Figure 3:**
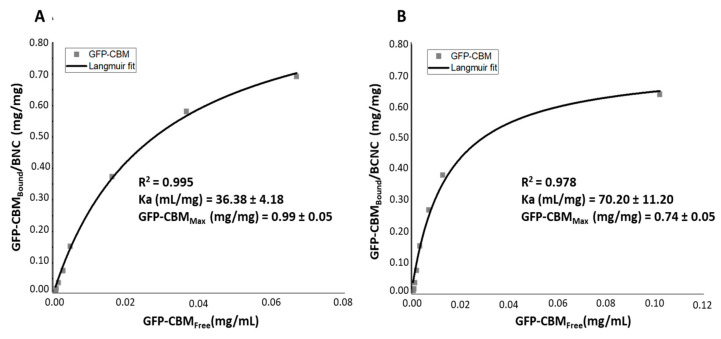
Adsorption isotherm of GFP–CBM onto: (**A**) BNC and (**B**) BCNC, after 2 h of incubation.

A GFP-fused carbohydrate-binding module (type 3a family [41], GFP–CBM3A) was selected to detect cellulose in biological tissues and to track its localization in histological slides. The GFP–CBM adsorption on BNC and BCNC was compared and the obtained data fitted to the Langmuir adsorption model [43,44]. Figure 3 shows the adsorption isotherms of GFP–CBM onto BNC and BCNC and the respective parameters of the Langmuir isotherm.

In both cases, the calculated R^2^ values were higher than 0.97, indicating a good fitting of the experimental data to the Langmuir isotherm model, as also demonstrated in other studies using type-A CBMs towards micro- and nano-crystalline cellulose, either from plants or bacterial [38,56,57,58,59].

**Figure 4:**
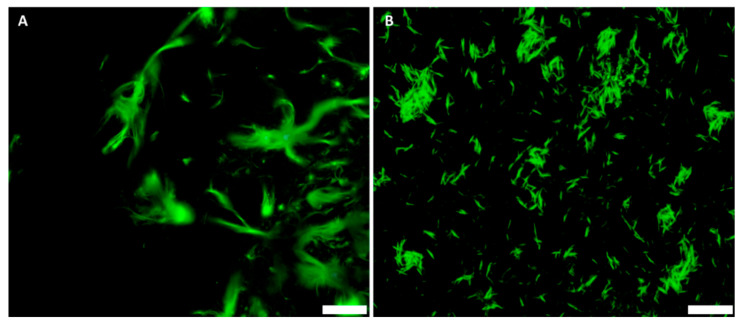
FM images of GFP–CBM (0.05 mg/mL) bound onto (**A**) BNC and (**B**) BCNC after 2 h of incubation. BNC and BCNC stock solutions were both at 0.25 mg/mL. Scale bar: 10 µm.

Regarding the Langmuir constant K_a_, BCNC (~70 mg/mL) show a higher value than BNC (~36 mg/mL), suggesting the GFP–CBM affinity is higher in the former case. The higher surface area of BCNC relative to BNC is probably responsible for the higher (apparent) affinity in the former case. However, the maximum adsorption was lower for BCNC (0.74 mg/mL for BCNC vs. 0.99 mg/mL for BNC). The analysis of the results obtained is not straightforward, as the two celluloses likely present different surface areas, as well as different surface charges. Several structural studies showed that GFP–CBM from the type 3A family, such as the one used in this work, bind to the hydrophobic face of crystalline cellulose, notably on the 110 face of crystallite [58]. Thus, the adsorption in higher amounts to BNC could be expected, since the surface charge on BCNC does not favor the interaction. With regards to the main purpose of this work, we could conclude that the interaction of the GFP–CBM with BCNC is not hampered by some surface sulfation associated with hydrolysis. Additionally, as shown in Figure 4, FM allows the visualization of the GFP–CBM adsorbed on hydrolyzed nanocellulose.

### 3.3. Considerations for Correlative Light Electron Micoscopy

In CLEM, FM observation is performed first, followed by TEM, because the electron beam can destroy the sample to some extent. The transition between FM and TEM must be made without dislocating the nanocrystals, a requirement strictly essential for any correlation to be possible. This proved to be challenging, since drying the sample before FM, to ensure the proper adhesion of the nanocrystals to the grid’s surface, resulted in a dramatic reduction of the fluorescence. On the other hand, using a moisturized/wet sample for FM analysis, followed by drying prior to TEM, resulted in the dislocation of BCNC on the surface.

These issues have been overcome by using gelatin, which provides both steric and electrostatic stabilization of colloidal suspensions [60,61,62]. Thus, BCNC were dispersed in gelatin, and a certain suspension volume was placed on the surface of the grid. Ahmad et al. (2011) demonstrated that silver nanoparticles synthesized in edible-grade gelatin showed a more homogenous size distribution compared to those produced by conventional methods; also, the nanoparticles had lower levels of aggregation in gelatin than the dried ones [63]. In a similar way, we hypothesized that, (i) in gelatin, the BCNC should remain well dispersed and spatially stabilized upon drying and (ii) the gelatin matrix would not interfere in the visualization of BCNC either in FM (e.g., due to autofluorescence) or in TEM (e.g., by exhibiting nanofibers with a complex shape that could hinder the detection of BCNC).

**Figure 5:**
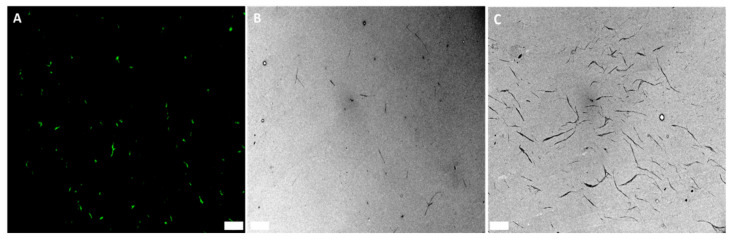
Microscopic images of GFP–CBM:BCNC (0.002 mg/mL) mixed with 2% gelatin (1:1) (**A**,**B**) and of GFP–CBM:BCNC aqueous suspension (**C**). (**A**) FM image. (**B**,**C**) TEM images. Scale bars: (**A**) 20 µm and (**B**,**C**) 1 µm.

As shown in Figure 5, the change of the dispersion matrix from water to gelatin resulted in well-dispersed GFP–CBM-labeled BCNC, as seen in FM (Figure 5A) and also in TEM (Figure 5B). While the dispersions of GFP–CBM:BCNC are stable, due to the sulfated groups, upon drying they tend to aggregate (Figure 5C), which is not the case when dispersed in gelatin (Figure 5B). Furthermore, in the latter, the calculated BCNC width (15 ± 4 nm) and length (449 ± 250 nm) were in accordance with the previous measurements obtained for BCNC in water suspensions (Figure 2C). Additionally, gelatin showed no autofluorescence, providing a suitable environment where nanocrystals exhibit high fluorescence. Thus, CLEM was performed using dispersions of GFP–CBM:BCNC in gelatin.

### 3.4. Correlative Light Electron Microscopy

**Figure 6:**
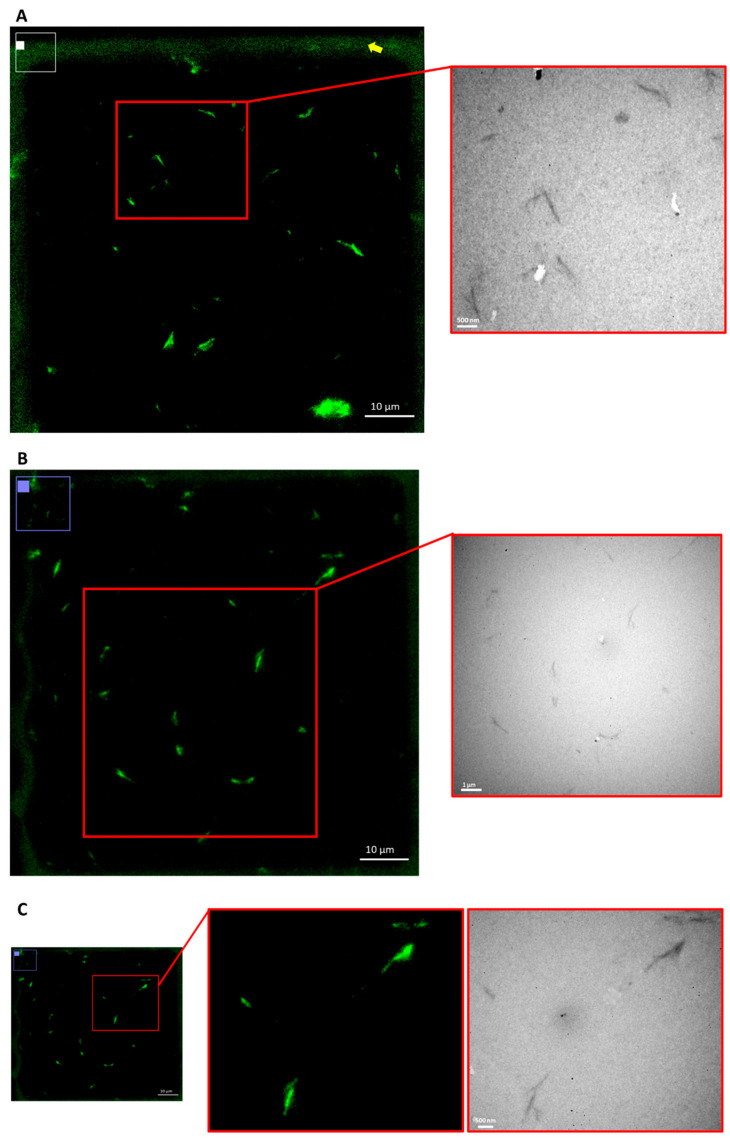
CLEM images of BCNC labeled with GFP–CBM. Small green dots in FM correspond to single BCNC in TEM (highlighted in the red boxes). The solid part of the TEM grid had some autofluorescence (yellow arrow); however, it did not interfere with the visualization as the GFP–CBM:BCNC-gelatin mixture was fixed in the transparent network mesh. Scale bars: (**A**–**C**) 10 µm for FM, (**A**,**C**) 500 nm and (**B**) 1 µm for TEM.

GFP–CBM: BCNC complexes were stabilized in gelatin, and a drop of the mixture was placed on a grid in order to assess the sensitivity of FM to detecting nanocrystals by CLEM. Given its lower resolution (limit ~200 nm [64]) compared to TEM, FM alone does not allow the measurement of the size of BCN crystals that give rise to the green signal detected (Figure 6), and it is not possible to reach conclusions on whether the fluorescent signal is generated by single crystals or by aggregates of several crystals. However, with CLEM imaging, it is possible to confirm the co-localization of the signals observed by FM and TEM, and, in several cases, the signal detected by FM seems indeed to correspond to single crystals, as detected by TEM. These results show that, despite the lower resolution limit compared to TEM, fluorescence imaging can be used as a feasible, robust and highly specific technique for the detection of isolated cellulose nanocrystals and thus also of larger fragments of BNC or smaller fibers released in vivo, in biological tissues.

### 3.5. In Vitro Assays

#### 3.5.1. Cytotoxicity Evaluation

Evaluating the biocompatibility of a material is an essential step toward its acceptance. Cell culture studies usually are the first step for the biocompatibility evaluation, as they are simple systems that minimize the effect of other variables. Although BNC is regarded as a biocompatible material [65], there are not so many reports on the cytotoxicity of BCNC, which we intend to use while studying the internalization (and subsequent detection) by macrophages. In this way, the cytotoxicity of BCNC was characterized, using both fibroblasts and macrophages.

**Figure 7:**
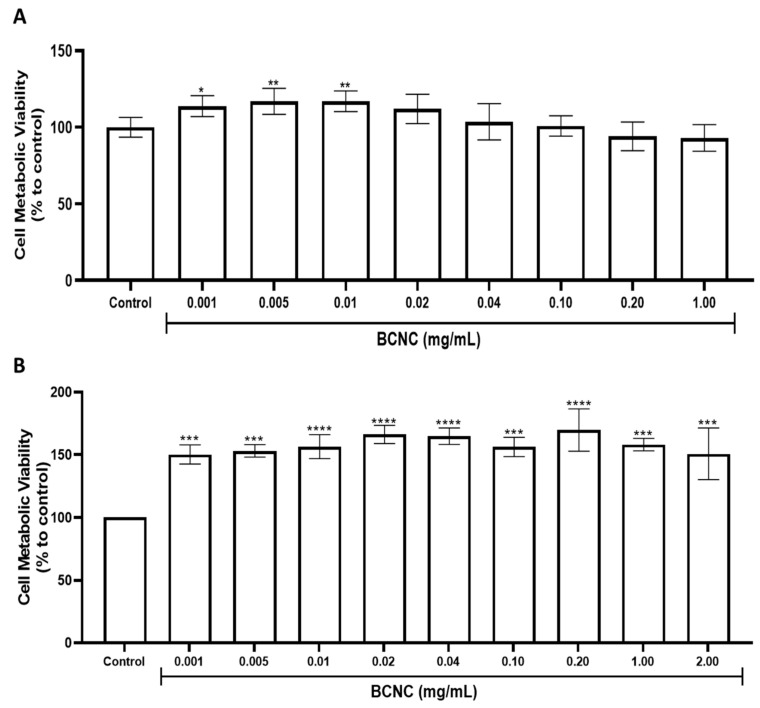
Metabolic viability of (**A**) L929 and (**B**) BMMΦ cells when cultured with increasing doses of BCNC for 24 h, as assessed by the Resazurin assay. PBS was used as the control. Data is expressed as a percentage relative to the control and is the mean ± SD of three independent experiments. All treatment conditions were compared with the control using Dunnett’s multiple comparison test. * *p* < 0.05, ** *p* < 0.01, *** *p* < 0.001 and **** *p* < 0.0001 compared to control (non-treated cells).

Results from the metabolic viability assay of L929 and BMMΦ cells when cultivated with BCNC for 24 h (Figure 7) show that BCNC did not significantly affect the viability of either cell type, even at very high concentrations (1 and 2 mg/mL, which are unlikely to be reached in vivo). The obtained data show that BCNC are not cytotoxic—as defined by ISO 10993, they did not reduce cell viability by more than 30%—consistent with reports from the literature [38]. However, a significant increase (by 50% on average) in the metabolic viability was obtained in BMMΦ cells after treatment with all tested concentrations. The effect was previously observed by others when exposing macrophages to vegetable nano and microfibrillated cellulose [66,67].

#### 3.5.2. Uptake of BCNC by BMMΦ Primary Cells

As in most tissues, gut-resident macrophages are important immune sentinels and effector populations. Positioned in close apposition to the epithelial layer, they are able to rapidly uptake and respond to any material breaching this barrier [68]. Thus, the process of the internalization of BCNC by macrophages was evaluated over time using GFP–CBM as described above.

**Figure 8:**
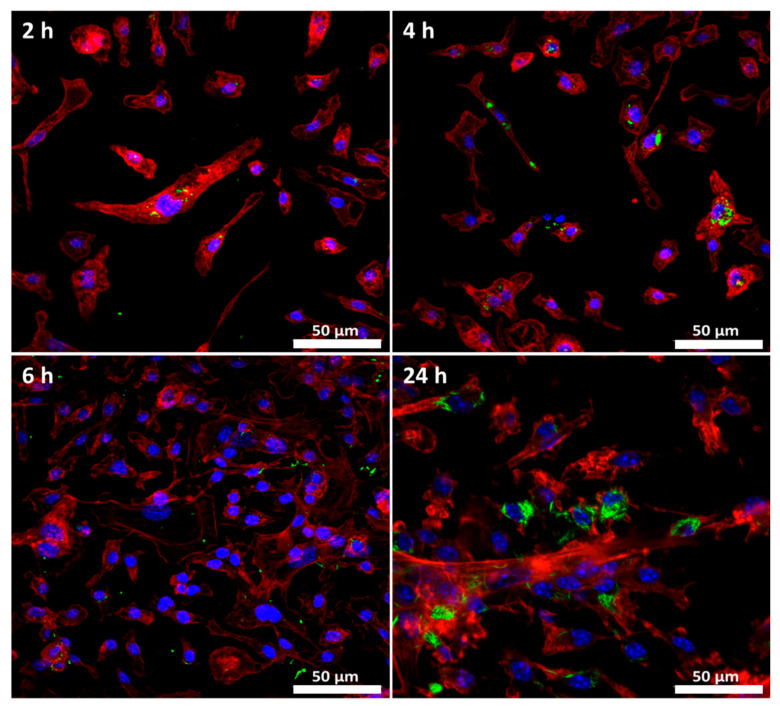
Cellular uptake of BCNC by phagocytic cells. FM images of macrophages exposed to 0.001 mg/mL BCNC for 2, 4, 6 and 24 h. DAPI (blue), Pha-red (red) and GFP–CBM (green) were used for nuclei, actin cytoskeleton and BCNC visualization, respectively. Scale bar: 50 µm.

Fluorescence images (Figure 8 showed that during short incubation periods (2 to 6 h), macrophages were already able to internalize BCNC; however, the amount of internalized material was very small. Despite this, it was possible to see labeled cellulose crystals inside some cells (most of the non-internalized BCNC was washed before cell fixation). At 24 h, clearly, a higher amount of the nanomaterial was uptaken. Several studies have reported that the macrophage internalization of foreign particles increases over time [69,70]. For instance, Erdem et al. (2021) showed that pristine cellulose nanocrystal uptake by alveolar macrophages increased significantly as the cell exposure period was longer (2, 6 and 24 h) [71]. Furthermore, we made an attempt to identify BCNC through ultrastructural analysis, which appear to be located within a lysosome (Supplementary Materials Figure S1). As described in the literature, macrophages are able to internalize particles with a diameter ranging from 6 to 6 000 nm [72]. Therefore, we demonstrate that, using fluorescent CBM, we are able to detect BCNC inside the macrophages, and thus, this may also be possible in vivo.

### 3.6. Nanocellulose Tracking in Animal Tissues

Despite several studies having reported important data supporting the safety of BNC as a food additive [33], its fate in the human body upon ingestion is still not totally clarified. Indeed, detecting small materials in biological tissues through imaging techniques is often hampered either by high background levels or by the lack of sensitive, nanomaterial-specific detection methods. Once it was demonstrated that fluorescence-based detection is a feasible method for the detection of nano-sized cellulose particles, a 21-day in vivo pilot study was made, to determine whether the same methodology was suitable for detecting micro- and/or nanocellulose in histological samples of animal tissues. It should be noted that UV + SBB treatment [48] eliminated the autofluorescence associated with this type of tissue, which, in several studies, made it difficult to apply fluorescence-based detection methods [73].

**Figure 9:**
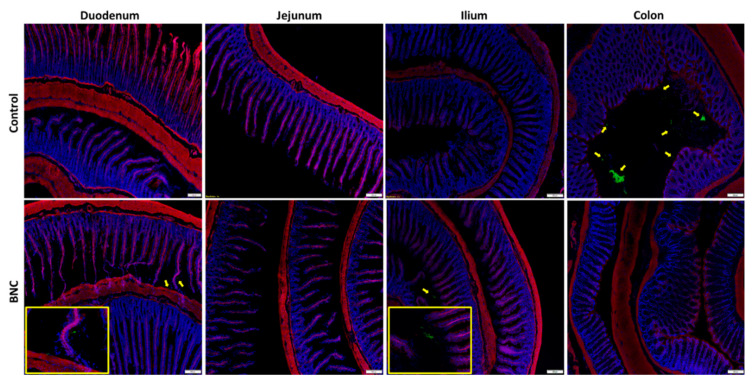
FM images of a male rat histological sample after a 21-day repeated-dose study. Four GIT regions (duodenum, jejunum, ilium and colon) were stained for nuclei (blue), actin cytoskeleton (red) and cellulose (green). Control (no gavage) and BNC (oral gavage) animals. Cellulose fibers were detected at the intestinal lumen or trapped between the villi (pointed out by the yellow arrow and the respective amplified image in the yellow box). Scale bar: 200 µm.

To better assess whether GFP–CBM-based detection is able to detect trace amount of cellulose along the GIT, the rats’ intestines were washed to remove, as much as possible, the cellulose present in the lumen and processed with SRT. Cellulose was found only in a few cases in the intestinal lumen or between the villi (Figure 9), without any notable difference in frequency or location among animals of different genders. However, no signs of the intestinal persorption of cellulose were found.

In fact, other studies examining the fate of cellulose particles in the intestinal tract have found no signs of translocation [26,33,74]. For instance, Mackie et al. (2019) sequentially studied the GIT fate of cellulose nanocrystal emulsions and their exposure to the intestinal mucus layer. They determined that the nanocrystals were clearly trapped in the intestinal mucosa, unable to reach the underlying epithelium, and were therefore considered safe emulsifiers [74]. Accordingly, the proposed routes of cellulose particle uptake predicted by others along the GIT, such as through M cells, the paracellular pathway and through enterocytes by transcytosis or passive diffusion [75], appear not to be supported since particles must first pass through the intestine mucus layer to subsequently interact with the gut wall. Our findings seem to confirm these reports, but a larger number of animals and a more detailed screening of the tissues must be performed to take more definitive conclusions.

## 4. Conclusions

A methodology for the specific detection of nano-scalar cellulose is critical for an understanding of its distribution in the human body. Herein, we demonstrate that fluorescence-based method can be used to detect and visualize different kinds of cellulose fibers, including nano-sized ones. The screening assay revealed simple, feasible and specific, allowing the detection of nanocrystals internalized in macrophages.

An exploratory work in vivo was performed, whereby no evidence of mucus layer translocation in the intestine was found. A more comprehensive study is required in order to take conclusions with regards to the potential cellulose persorption and thus contribute to the analysis of the safety of BNC as a food additive.

## Data Availability

The relevant data for the discussion are completely supplied in the Results and Discussion section. Raw data are available upon request.

## Supplementary Materials

The following supporting information can be downloaded at: www.mdpi.com/article/10.3390/nano12152605/s1, Figure S1: Uptake of BCNC by BMMΦ primary cells—Electron Microscopy.
