# Supplementary material for: Tracking Bacterial Nanocellulose in Animal Tissues by Fluorescence Microscopy
Source: Nanomaterials (Basel). 2022 Jul 28;12(15):2605. doi: 10.3390/nano12152605 (PMC9370207; doi:10.3390/nano12152605)
Supplement: Supplementary file 1 [file nanomaterials-12-02605-s001.zip › nanomaterials-1815211-supplementary.pdf]

Supplementary Material

Tracking Bacterial NanoCellulose in animal tissues by fluorescence microscopy

Renato Mota 1,2, Ana Cristina Rodrigues 1,2, Ricardo Silva-Carvalho 1,2, Lígia Costa 1,2, Daniela Martins 1,2, Paula Sampaio 3,4, Fernando Dourado 1,2 and Miguel Gama 1,2,*

1 CEB - Centre of Biological Engineering, University of Minho, Campus de Gualtar, 4710-057 Braga, Portugal;

2 LABBELS - Associate Laboratory, 4710-057 Braga, Portugal;

3 i3S - Instituto de Investigação e Inovação em Saúde, Universidade do Porto, Rua Alfredo Allen 208, 4200-135 Porto, Portugal;

4 IBMC - Instituto de Biologia Molecular e Celular, Universidade do Porto, Rua Alfredo Allen 208, 4200-135 Porto, Portugal;

*** Corresponding Author:**

Miguel Gama

fmgama@deb.uminho.pt

Department of Biological Engineering

University of Minho

Campus de Gualtar

4710-057 Braga

Portugal

Supplementary Data

Uptake of BCNC by BMMΦ primary cells – Electron Microscopy

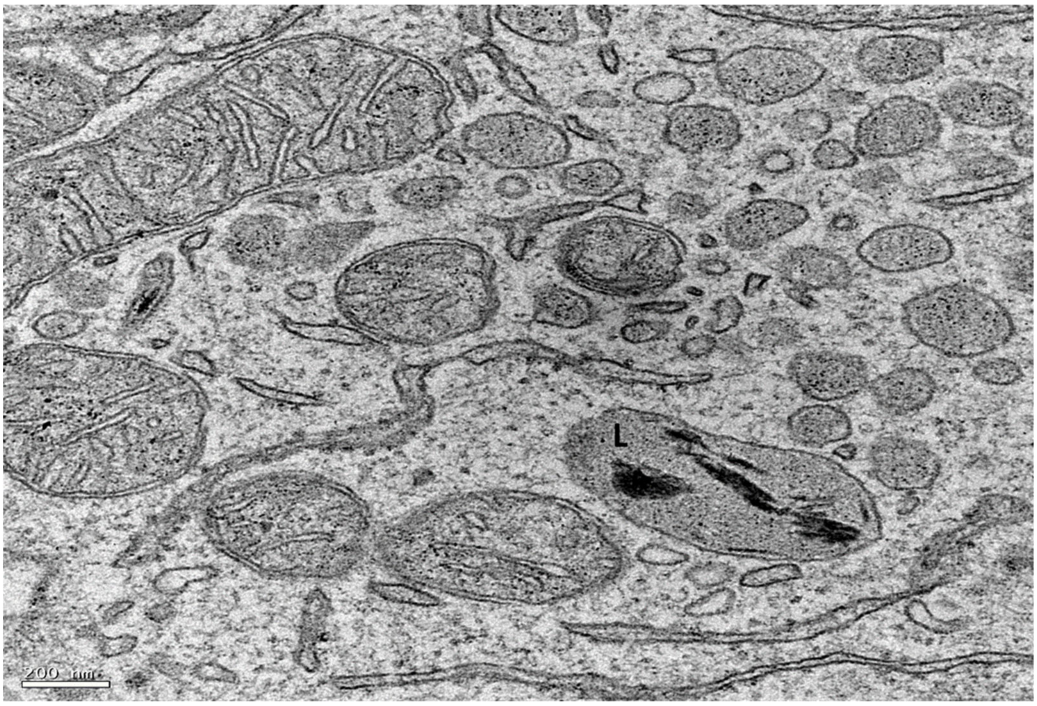

Figure S1. Cellular uptake of BCNC by phagocytic cells. TEM image of macrophages exposed to 0.001 mg/mL BCNC for 4h. Possible lysosome (L) containing previously phagocytized BCNC. Scale bar: 200 nm.
